# A Case Report: Monoclonal Gammopathy of Undetermined Significance Presenting With Renal Amyloidosis and Reactive Thrombocytosis

**DOI:** 10.7759/cureus.59580

**Published:** 2024-05-03

**Authors:** Nyein Wint Yee Theik

**Affiliations:** 1 Internal Medicine, Memorial Healthcare System, Pembroke PInes, USA

**Keywords:** immunofixation, serum electrophoresis, renal al amyloidosis, plasma cell disorder, monoclonal gammopathy of undetermined significance (mgus)

## Abstract

Monoclonal gammopathy of undetermined significance (MGUS) is a pre-neoplastic condition involving plasma cells or lymphoplasmacytic proliferation-clinical presentations ranging from asymptomatic to symptoms of systematic organ involvement. It is commonly associated with light chain amyloidosis and usually strengthens during diagnosis. It is usually associated with thrombocytopenia in addition to anemia. This case report focuses on a 63-year-old Hispanic female who initially presented without any symptoms but with severe thrombocytosis. The finding of renal amyloidosis boosts the final diagnosis of MGUS. Rarely a patient is initially presented with severe thrombocytosis despite thrombocytopenia, which is commonly associated with plasma cell disorders. The case emphasizes the need for considering plasma cell disorders in patients with abnormal hematologic manifestations. It highlights the importance of timely identification and intervention to prevent irreversible organ damage by providing a detailed analysis of the clinical presentation and diagnostic evaluation.

## Introduction

Monoclonal gammopathy of undetermined significance (MGUS) is distinguished by a pre-neoplastic condition involving plasma cells or lymphoplasmacytic proliferation. This disorder exhibits similarities with multiple myeloma (MM), Waldenstrom macroglobulinemia (WM), and amyloid light chain amyloidosis (AL). Its diagnostic criteria include a serum monoclonal protein (M protein) concentration of <3 g/dL, bone marrow (BM) with a monoclonal plasma cell percentage of 10 percent, and the absence of specific organ impairments, such as lytic bone lesions, anemia, hypercalcemia, renal insufficiency, and hyperviscosity [1].

MGUS is further categorized into three subtypes based on the M protein isotype: immunoglobulin M (IgM) MGUS, non-IgM including IgG, IgA, or IgD MGUS, and light-chain MGUS (LC-MGUS) [2]. Among these, non-IgM MGUS is the most prevalent subtype. It can progress to smoldering MM, symptomatic MM, or less frequently to AL amyloidosis, light chain deposition disease, or other lymphoproliferative disorders [3]. MGUS is associated with a spectrum of health-related complications, including fractures, infections, neuropathies, thrombosis, and increased mortality [4]. Its manifestations can range from systemic disorders affecting multiple organ systems to those primarily affecting specific organs such as the kidney, nervous system, or skin [5].

While renal amyloidosis is a relatively rare complication of MGUS, it can lead to severe kidney dysfunction due to the deposition of abnormal protein aggregates in the renal parenchyma [6]. This case report represents a unique instance where MGUS manifested as renal amyloidosis and reactive thrombosis. This finding underscores the importance of considering plasma cell disorders in patients exhibiting atypical renal and hematologic manifestations and raises questions regarding the underlying pathophysiological connections. Timely identification and intervention are crucial to prevent irreversible organ damage and enhance patient survival.

This case report aims to provide a comprehensive analysis of the clinical presentation, diagnostic evaluation, and management strategies employed in a patient with renal amyloidosis and reactive thrombocytosis secondary to MGUS. Subsequent sections will discuss the patient's clinical history, laboratory findings, diagnostic procedures, and therapeutic interventions, shedding light on the complexities of managing such cases.

## Case presentation

The patient, a 63-year-old Hispanic female, sought evaluation by the hematology-oncology outpatient service due to abnormal findings on a complete blood count (CBC), including leukocytosis and thrombocytosis, with a white blood cell count of 17 x 10^3^/UL and platelet count of 905 x 10^3^/UL. She had a medical history notable for gout, hypertension, and hyperlipidemia. During the initial evaluation, the patient reported no symptoms such as unintentional weight loss, fevers, night sweats, flushing, or lymphadenopathy.

A CBC conducted three months earlier had shown normal platelet counts with mild leukocytosis with a white blood cell count of 12 x 10^3^/UL. As a possible explanation for leukocytosis, the patient had been taking a daily dose of 600 mg of allopurinol for a recent gout flare-up. Due to worsening platelet count despite improving leukocytosis on the following assessment, which is more than 1000 x 10^3^/UL, the patient was initiated on a daily dose of hydroxyurea at 500 mg, subsequently increased to 1000 mg daily for severe thrombocytosis. Extensive testing for myeloproliferative neoplasms (MPN), including Janus Kinase 2 (JAK2), Myeloproliferative leukemia Virus Oncogene (MPL), Calreticulin (CALR), and B-cell leukemia/Lymphoma-Abelson Murine Leukemia Viral Oncogene Homolog 1 (BCL-ABL), all returned negative results. Moreover, OnkoSight advanced next-generation sequencing (NGS) myeloid panel testing also yielded negative for any myeloid neoplasm.

**Figure 1:**
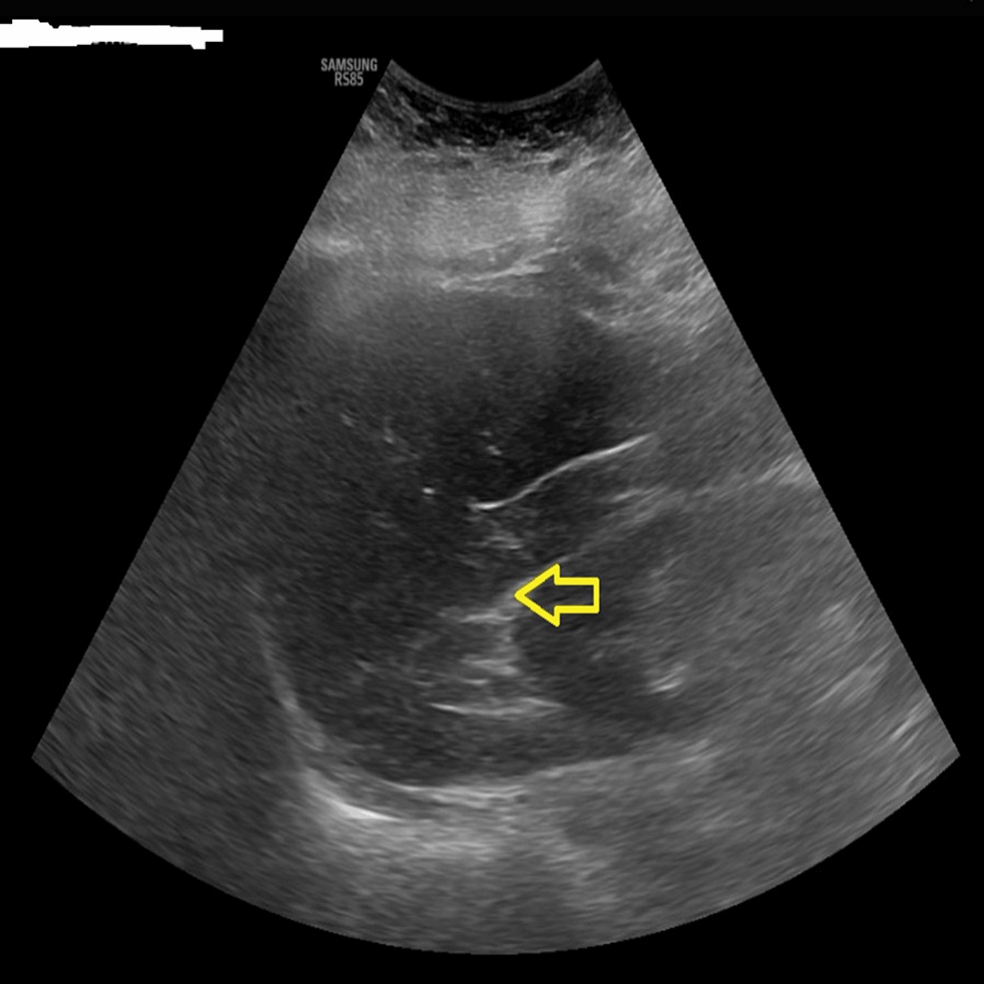
Splenic ultrasound showing negative for structural splenic abnormalities

Common potential causes of reactive thrombocytosis, such as anemia, infection, post-splenectomy, or functional asplenia, have been systematically ruled out through diagnostic measures (Figure 1). A non-specific finding in a spleen ultrasound, normal levels of iron and ferritin, and hemoglobin levels within the expected range all contribute to this comprehensive assessment. Furthermore, an infection workup has yielded negative results. A mild elevation in sedimentation rate with a value of 45 mm/hr noted during the initial evaluation was attributed to a recent gout flare. However, reassuringly, it trended back to within normal limits in subsequent follow-up laboratory tests. The patient has no suspicions of any autoimmune disorder despite her having a gout flare around the same time but no symptoms at that time. The results of the main laboratory testing are summarized in Table 1.

**Table 1:** Main laboratory data WBC: White Blood Cell, Hb: Hemoglobin, Plt: Platelet, ESR: Erythrocyte Sedimentation Rate.

Laboratory findings	Initial Result	Follow Up Result	Reference range
WBC (10^3^ /uL)	17	12	3.8-11
Hb (g/dl)	13	13.5	11.5-15.5
Plt (10^3 ^/uL)	905	> 1000	150-400
ESR (mm/hour)	40	45	0-20

Bone Marrow biopsy does not reveal any morphologic evidence for MPN, blast elevation, or gross abnormalities of megakaryocytes in the marrow. However, it revealed that for 10% of plasma cells, polytypic with some kappa and excess lambda, possibilities of plasma cell neoplasm in addition to focal small apple-green birefringence in a blood vessel wall by Congo red stain suggestive of focal amyloidosis. Despite the lab and bone marrow biopsy result, the International Prognostic Score of Thrombosis for ET (IPSET-Thrombosis) risk score was collected as 2 points where the patient is put with the possible early-stage triple-negative MPN with intermediate risk, which is defined by the lack of JAK2, CALR, or MPL mutations and found in about 10% of thrombocytosis patients.

Additionally, serum protein electrophoresis (SPEP) showed beta-2 possible monoclonal protein with gamma possible monoclonal protein, which boosts the possibility of plasma cell dyscrasias with low albumin level. Subsequently, a serum immuno-fixation test was also detected for IgG Lambda monoclonal protein. However, lab findings such as the elevation of IgA rather than IgM and the elevation of free kappa in addition to lambda, which leads to a normal kappa/lambda ratio, are very uncommon in order to diagnose multiple myeloma.

**Figure 2:**
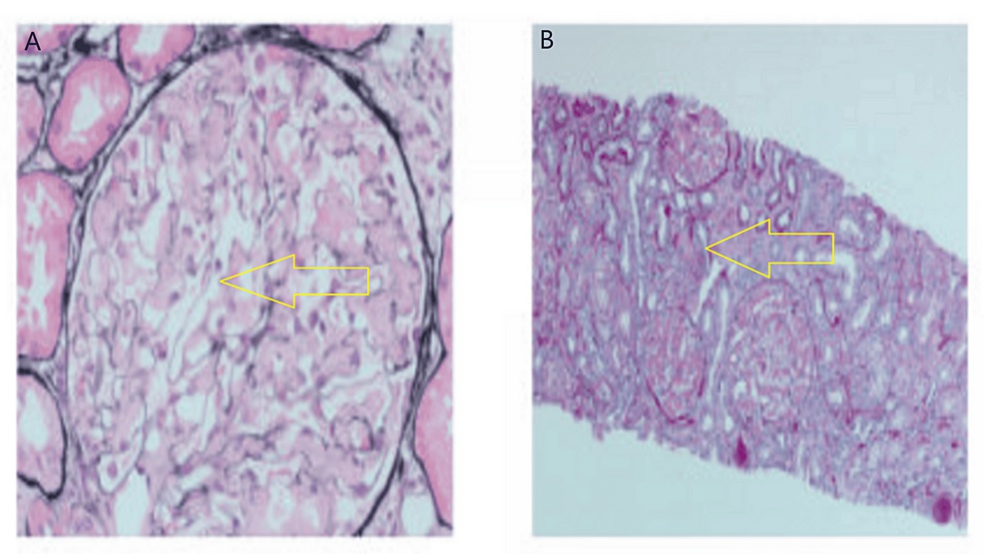
Diffuse mesangial expansion by amorphous acellular and focal interstitial amyloid deposition (A) Amorphous and acellular material (amyloid) within glomeruli as shown by yellow arrow (Periodic acid-silver methenamine stain), (B) Pale amorphous material and acellular material (amyloid) within glomeruli as shown by yellow arrow (periodic acid-Schiff stain).

In addition to the aforementioned hematological lab findings, nephrotic range proteinuria was noted. The results of the renal function and other laboratory testing are summarized in Table 2. As AL-amyloidosis is a common manifestation of plasma cell dyscrasia, renal biopsy was performed and consistent with amyloid disease (Figure 2). Additional renal amyloidosis findings plus bone biopsy findings lead us to finalize the possible diagnosis of IgG-L-MGUS.

**Table 2:** Other laboratory data BUN: Blood Urea Nitrogen, AST: Aspartate Transaminase, ALT: Alanine Transaminase, LDL: Low-Density Lipoprotein, HDL: High-Density Lipoprotein, RBC: Red Blood Cell.

Laboratory findings	Initial results	Reference range
Comprehensive Metabolic Panel		
BUN (mg/dL)	40	6-25
Creatinine (mg/dL)	1.88	0.55-1.02
Albumin (g/dL)	2	3.4-5.4
AST (U/L)	12	0-34
ALT (U/L)	10	6-29
Total bilirubin (mg/dL)	0.2	0.3-1.2
Lipid Panel		
Total Cholesterol (mg/dL)	212	<200
LDL (mg/dL)	112	<130
HDL (mg/dL)	72	>50
Urinalysis		
Gross blood	Negative	Negative
Protein level (mg/dL)	>3000	Negative
RBC (/hpf)	2	0-4
Microalbumin (mg/dL)	>114	0.0-1.8
Protein/creatinine ratio	>30,000	<0.15
Albumin/creatinine ratio (mg/g)	>500	0.0-29.9

## Discussion

Thrombocytopenia is a joint presentation in patients with plasma cell dyscrasias. Pathogenesis is typically due to bone marrow replacement by the transformed cells or follows treatment with chemotherapy [7]. Our presented case demonstrates a rare CBC finding like thrombocytosis where the patient also developed renal amyloidosis, which boosts the confirmation of MGUS diagnosis. Additionally, serum protein electrophoresis (SPEP) and immuno-fixation tests indicated monoclonal protein abnormalities. However, the atypical elevation of IgA and the kappa/lambda ratio made diagnosing multiple myeloma less likely. Also, the conclusion of the final diagnosis involved ruling out MPN through extensive testing, including JAK2, MPL, CALR, and BCL-ABL. This case presentation also highlighted the complete diagnostic workup for thrombocytosis to rule out the most common causes of reactive thrombocytosis, such as anemia, infection, or splenic abnormalities.

Among the screening tools for monoclonal protein associated with AL amyloidosis, serum free light chain assay has the highest sensitivity, followed by serum immunofixation electrophoresis (SIFE) and SPEP [8]. Due to high sensitivity, these tests are usually used as initial screening tests before bone marrow biopsy. Our patient presented with nephrotic range proteinuria; subsequently, renal biopsy was positive for amyloidosis. The unique hematological finding and renal biopsy results led to the diagnosis of IgG-L-MGUS, highlighting the essential and rare finding in diagnosing other plasma cell disorders.

## Conclusions

In the future, this case presentation will shed light on the complexity of diagnosing and atypical presentation of MGUS and other plasma cell disorders, emphasizing the importance of a thorough and multidisciplinary approach. The case also emphasizes recognizing plasma cell disorders in patients with unusual hematological and renal manifestations. The patient’s journey starts from the initial asymptomatic presentation and hematological finding to the final diagnosis, which requires a comprehensive evaluation and thorough diagnostic workup. The detailed underlying mechanism of action for the association between MGUS and reactive thrombocytosis is questionable. Clinical findings will represent the imperative nature of timely identification and intervention to prevent irreversible organ damage. Hopefully, they will help improve the survival of patients with MGUS and its related complications in the future.
